# The Effects of the COVID Pandemic on Patients with IBD: Lessons Learned and Future Directions

**DOI:** 10.3390/jcm11237002

**Published:** 2022-11-27

**Authors:** Eva Zhang, Britt Christensen, Finlay Alistair Macrae, Rupert Leong

**Affiliations:** 1 Department of Medicine, Macquarie University, Macquarie Park 2113, Australia; 2 Department of Gastroenterology, Royal Melbourne Hospital, Melbourne 3050, Australia; 3 Department of Medicine, University of Melbourne, Melbourne 3050, Australia; 4 Department of Colorectal Medicine, Royal Melbourne Hospital, Melbourne 3050, Australia; 5 Department of Gastroenterology, Concord Repatriation General Hospital, Concord 2137, Australia

**Keywords:** inflammatory bowel disease, SARS-CoV-2, COVID-19, risk, vaccination, infection

## Abstract

The COVID-19 pandemic has caused extended global disruption and changed healthcare behaviour and delivery in patients with inflammatory bowel disease, many of whom take immune modifying treatment. Although there were fears about the vulnerability of IBD patients to SARS-CoV-2 infection, we have learnt that overall IBD patients are equivalent to the general population in both viral acquisition and infection outcomes. Overall IBD patients obtain effective vaccine-induced immune responses, although in some groups an additional vaccine dose is required to constitute a primary course. The pandemic has led to significant changes in healthcare delivery, some of which will be enduring. As we grapple with the challenges of recovery, the lessons learnt will continue to be important in optimising outcomes in future outbreaks.

## 1. Introduction

The SARS-CoV-2 viral pandemic has had devastating health consequences and has led to global disruption. There were significant concerns in patients with inflammatory bowel disease (IBD) who were potentially vulnerable due to disease activity and the immune modifying medications administered. This led to the urgent need to evaluate the risk of viral acquisition in IBD, the impact of IBD medication on infection outcomes, and vaccine-induced immune response. Patients were also faced with significant changes in the delivery of healthcare. Here we review some of the lessons we have learnt, as virus variants continue to evolve. 

## 2. Acquisition

SARS-CoV-2 is a single-stranded RNA virus that enters cells via the angiotensin converting enzyme 2 (ACE2) which is highly expressed in lung and intestinal tissue. Although primarily a respiratory illness, the SARS-CoV-2 infection which is also termed COVID-19 syndrome, often causes gastrointestinal symptoms. This led to initial concerns that patients with inflammatory bowel disease (IBD) who may already have compromised integrity of the intestinal barrier, were at higher risk of infection and more severe disease [1]. This was magnified by the potential for the deleterious effects of immunosuppressive medications in use to control bowel inflammation. These concerns was reflected in the risk matrix published by the 2020 British Society of Gastroenterology Guidelines which stratified IBD patients into low, moderate, and high risk [2] based on medication regimen and comorbidities. However, these recommendations have since been withdrawn [3] as our understanding of risk, virus variants, and public health intervention evolves. 

A systematic review and meta-analysis shows IBD patients overall are not at increased risk of SARS-CoV-2 infection compared to the general population [4]. There were initial concerns about an increased risk of infection and adverse outcomes with sulfasalazine and 5-ASA in both IBD and rheumatological patients [5]. The impairment of type I IFN production and thus effective immune responses to infection, induced by sulfasalazine were posed as a possible reason why this was so [6]. However, subsequent data from the SECURE-IBD registry, a large international cohort registry, has not supported this finding [7]. Patients on advanced therapies including anti-TNF, ustekinumab, vedolizumab, and JAK inhibitors are not at increased risk of infection [4]. 

## 3. The Impact of IBD Medications on Outcomes of COVID-19 Infection

Overall, severe outcomes and mortality from COVID-19 infection are similar in IBD patients compared to the general population [4]. Like the general population, older age and the presence of multiple comorbidities increased the risk of severe outcomes. In the analysis of outcomes stratified by IBD medications, systemic corticosteroids are associated with increased odds of severe COVID-19, hospitalisation, and death [7]. However anti-TNF monotherapy, IL12/23 antagonists, and tofacitinib are associated with reduced odds of hospitalisation, possibly through abrogation of the cytokine storm induced with severe COVID-19 infection (Table 1). Methotrexate is marginally associated with an increased odds of hospitalization, but not severe infection nor death [7]. Analysis of data from the SECURE-IBD registry suggests anti-TNF antagonist and thiopurine combination therapy is associated with a significantly increased risk of hospitalization or death aOR 1.8 (95% CI 1.26–2.62) but not severe COVID-19 infection. This association was not demonstrated in those on anti-TNF combined with methotrexate. Active IBD is also associated with worse outcomes from COVID-19 infection. In patients ≤50 years, severe disease as defined by physician global assessment was independently associated with need for ventilation, death and hospitalisation secondary to COVID-19. This was not the case in those >50 years, probably due to advanced-age being a more significant driver of risk of severe infection compared to disease activity [8]. 

## 4. Changes in the Delivery of Healthcare

The pandemic has changed the way we deliver healthcare. A focus on personal protective equipment (PPE), masks, hand hygiene, and social distancing were made [1]. Borders were closed and lockdowns imposed to prevent viral transmission. Individuals were advised to avoid unnecessary visits to medical facilities [2], including the restriction of visitors for hospitalised patients. This had major implications on face-to-face appointments, emotional support for inpatients, infusion visits, and elective procedures.

### 4.1. Endoscopies 

Endoscopic procedures were significantly diminished from 2020–2021 due to concern about viral transmission via aerosol droplets, in addition to the diversion of health utilisation away from elective procedures [9]. Risk stratification during this period suggested that endoscopy should be undertaken for the most urgent cases including confirmation of a new diagnosis, severe acute ulcerative colitis, or partial bowel obstruction [10]. As endoscopy units resume usual operating capacities, there remains a huge backlog of surveillance procedures. This has important implications not only for disease monitoring, but also for cancer surveillance in IBD [11]. To assist triaging IBD surveillance procedures in the recovery period, the British Society of Gastroenterology (BSG) suggest risk stratification using faecal occult blood test in combination with faecal calprotectin. Further, those at highest risk of dysplasia should be prioritised, including those with primary sclerosing cholangitis (PSC), previous IBD-related dysplasia, and severe disease [12]. There is no data to date, quantifying the impact of the pandemic on the on the diagnosis and treatment of colorectal cancer. However, the rate of colorectal cancer diagnoses fell significantly during the pandemic and thus there is significant concern about pandemic-related delayed detection of cancers and timely treatment.

### 4.2. Telemedicine, Non-Invasive Disease Monitoring, and Point of Care Tests 

One of the most significant changes the pandemic triggered of note, was the rapid shift to telemedicine to limit exposure to healthcare settings [9]. Telemedicine, coupled with objective measures of disease activity has proven to be effective and safe in the care of IBD patients, receiving high patient satisfaction [13]. Due to the limitations surrounding endoscopic procedures, there was a greater reliance on non-invasive methods of disease assessment, including faecal calprotectin and intestinal ultrasound. The use of point of care testing for drug levels and faecal calprotectin at home [14] was also introduced. Despite easing and removal of restrictions, the demand for telemedicine and non-invasive disease assessment remains high. They will continue to play a major role in the future delivery of healthcare and remain suitable for a significant proportion of IBD patients. 

### 4.3. Switching from Intravenous to Subcutaneous Therapies 

IBD patients on intravenous (IV) infliximab or vedolizumab were offered to transition to subcutaneous (SC) therapies in select centres during the pandemic [15]. The rationale was to avoid unnecessary exposures that travelling to infusion centres would pose. The transition to subcutaneous therapies continues to expand worldwide, although COVID-19 is no longer a significant factor in its uptake [16]. 

## 5. Immune Response to Vaccination

Vaccination has been integral in combating the pandemic. The impact of IBD medications on vaccine-induced immune response, and the natural course of waning immunity in all groups has been a field of intense scrutiny. Infliximab attenuates antibody responses after second dose vaccination compared to vedolizumab and the general population [17]. Combination therapy with infliximab and immunomodulator magnify the deleterious effect. Infliximab levels have no correlation to antibody responses, and the timing of vaccination in relation to infliximab administration bore no relevance to antibody concentrations [18]. Corticosteroids and JAK inhibitors also impair antibody responses [19,20]. This led to the advice for anti-TNF and corticosteroid treated patients to obtain a third vaccine dose to complete their primary vaccine course [21]. However, ustekinumab and vedolizumab treated patients achieve equivalent antibody responses compared to the general population (Table 2). Further reassuringly, overall most IBD patients achieve seroconversion after two doses of approved SARS-CoV-2 vaccines [22]. T cell responses are unpaired from antibody responses [20,23]. Approximately 20 percent of IBD patients and healthy controls do not mount a T cell response after 2 dose vaccination [23]. Interestingly anti-TNFs are associated with augmentation of T cell responses, although the biological mechanism behind this is unclear [24,25]. Tofacitinib is associated with diminished T cell responses [26], whilst ustekinumab and vedolizumab are associated with equivalent responses to the general population. 

### Omicron and Booster Vaccines

As vaccine-induced antibody titres fall with time, boosters were introduced worldwide [27]. In IBD patients, boosters lead to an upsurge in antibody concentration, however anti-TNFs continue to be associated with attenuated responses compared to other advanced therapies and the general population [28]. Evolving virus variants also sparked fresh concerns, with Omicron B.1.1.529 declared a variant of concern in November 2021 [29]. The original vaccines were developed with the spike protein of the ancestral strain as the target; however Omicron possesses over 30 mutations in the spike region rendering the neutralising antibody activity of vaccines less efficacious. This led to rising cases of infection worldwide and triggered renewed lockdowns worldwide. Infliximab-treated patients are more likely to report breakthrough infection compared to vedolizumab-treated patients in this setting [28]. However T cell responses are preserved as it is targeted at multiple epitopes preserved across viral variants [30]. Further, bivalent booster vaccines are now available which deliver greater neutralising activity against the Omicron variant to provide greater protection [31]. This means timely boosters should be prioritised, particularly those who are on anti-TNFs, JAK inhibitors, and high dose systemic corticosteroids who have diminished antibody responses.

## 6. Vaccine Uptake Amongst IBD Patients 

**Scheme 1:**
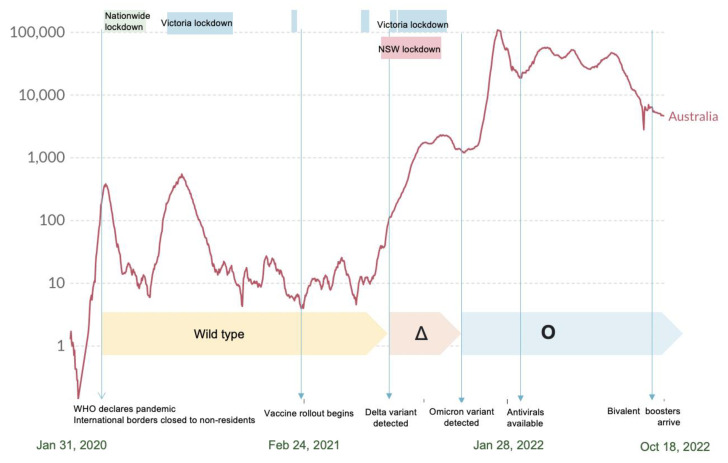
Daily COVID-19 cases (Log) in Australia, adapted from [39].

Vaccination has been a key strategy in the pandemic, however vaccine uptake was lukewarm at first, despite the availability of vaccines. In the context of the rapid development of vaccines, concerns about long term side effects were prominent [32]. The exclusion of immunocompromised individuals from the original vaccine trials and the paucity of data specific to IBD patients at this time magnified the uncertainty. In a North American survey conducted within two months of the vaccine rollout, vaccine intent was 60% amongst IBD patients in the community [33]. In particular, the potential to trigger an IBD flare was a prominent concern. Misconceptions about vaccination triggering an IBD flare, and the safety of vaccination in pregnancy were also found to be negative predictors of vaccine uptake [34]. In Australia, vaccine uptake was high partly due to the delayed vaccine rollout compared to North America and Europe, allowing time to observe the impact of vaccination in other countries (Scheme 1).

Registries and observational studies confirm that IBD patients are not at higher risk of vaccine side effects compared to the general population. This includes gastrointestinal manifestations [35], hence negating the misconception of vaccine induced IBD flares. In fact, those on advanced therapies are less likely to experience side effects compared to the general population, possibly due to downregulation of inflammatory cytokines. Reassurance from health providers and the global experience with vaccination has assisted in uptake with time. 

Boosters now remain the focus as restrictions ease and the rates of infection globally remain above 500,000 per day [36]. A survey of 449 IBD patients at an infusion centre in the United Kingdom revealed that 45% had received three doses, and 43% had received four doses [37]. Younger age was associated with lower booster vaccine uptake, with only 29% of those aged 30 years or younger having received four doses. These findings relate to the easing of global concern surrounding SARS-CoV-2, reduced health messaging, and the perceived lower risk of severe disease in younger people [38]. 

## 7. Considerations around COVID-19 Infection in IBD Patient

In IBD patients who contract COVID-19 infections, two main considerations need to be made; firstly, the management of IBD medications, and secondly, the need for antiviral therapies. Firstly 5-ASA and topical therapies can be continued without interruption [40]. Given the association between systemic corticosteroids and adverse outcomes in COVID-19 infection, those on corticosteroids should have therapy tapered and weaned, or switched to budesonide, if practicable. However in those who are critically ill from COVID-19 infection, IV dexamethasone has a role in improving outcomes [41]. Methotrexate, thiopurines, biologics and small molecules can be withheld for 10–14 days, until symptom resolution. However, it is important to note that these are guidelines only. These medications are often continued in practice because of asymptomatic or mild COVID-19 infection, or because clinicians are not informed until sometime after COVID-19 infection. Continuation of IBD therapy may be more important in patients who have brittle control of their IBD. In the setting of a severe acute flare, systemic corticosteroids may be unavoidable. Certainly severe IBD is associated with worse COVID-19 outcomes [8]. 

Patients on advanced therapies who have symptomatic infection have several antiviral options provided they do not require oxygen and remain at home. Molnupiravir, nirmatrelvir/ritonavir (Paxlovid**^®^**) are oral options [42]. Sotrovumab is an intravenous option. It is important to be aware of CYP3A4 drug-drug interaction with nirmatrelvir/ritonavir (Paxlovid**^®^**^)^. This is relevant for IBD patients who are on JAK inhibitors and calcineurin inhibitors. Tofacitinib and upadacitinib should be paused and commenced 3 days after completing nirmatrelvir/ritonavir [43]. The coadministration of ciclosporin and nirmatrelvir/ritonavir should be avoided due to challenges in dose adjustment and monitoring drug levels. However if necessary, the total daily dose of ciclosporin should be reduced by 80 percent with ciclosporin level assessment on day 6. There are no expected interactions with other IBD medications including anti-TNF, ustekinumab, vedolizumab, ozanimod, thiopurines or 5-ASA. In those who require hospitalisation, remdesivir, or baricitinib may also be indicated with IV dexamethasone.

## 8. The Interaction of the Gut Microbiome with Infection and Vaccination

The gut microbiome is recognised to modulate mucosal immune regulation and may play a role in vaccine induced immune responses. Both SARS-CoV-2 vaccination and infection induce alterations in the intestinal microbiome. In an observational study, BNT162b2 recipients who had surrogate viral neutralising test (sVNT) inhibition of less than 60% had a low level of Actinobacteria, particularly B. adolescentis [44]. A higher relative abundance of bacteria with flagella in the baseline gut microbiome correlated with higher antibody responses to BNT162b2 vaccines. SARS-CoV-2 infection in hospitalised, antibiotic naïve patients also induced changes in the microbiome that persisted after symptom resolution, including a higher number of opportunistic pathogens including *Clostridium hathewayi, Actinomyces viscosus,* and *Bacteroides nordii* compared to controls [45]. In IBD patients where gut dysbiosis is a driver of disease itself [46], these effects of SARS-CoV-2 infection and vaccination on the microbiome have not been examined to date.

## 9. Conclusions

**Figure 1:**
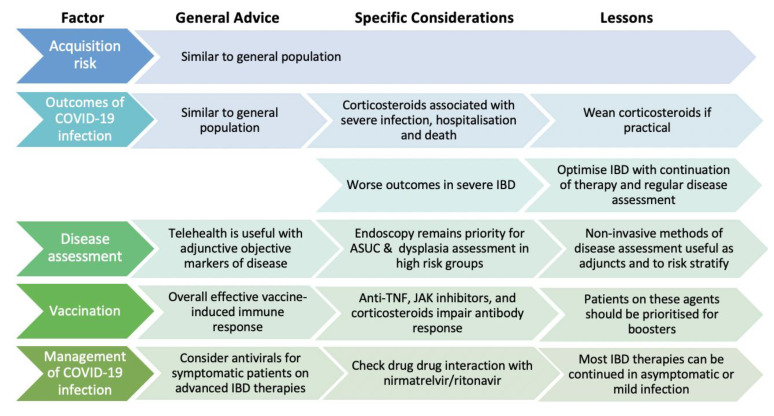
Considerations in the care of inflammatory bowel disease patients surrounding the SARS-CoV-2 pandemic.

Public health interventions including vaccination, restrictions, and a change in the delivery of healthcare have helped IBD patients and clinicians navigate the pandemic over the past two years. Overall IBD patients are not at increased risk of acquisition, nor of severe outcomes compared to the general population (Figure 1). Infection causes changes in the microbiome and often leads to transient gastrointestinal symptoms. The vaccine-induced immune response is effective, although those on anti-TNF, JAK inhibitors, and high dose corticosteroids have attenuated humoral responses. Parodically anti-TNF monotherapy is associated with augmentation of cellular responses. Systemic corticosteroids should be tapered if possible, and advanced therapies withheld in the case of significantly symptomatic COVID-19 infection. However, this decision should be made on a per-patient basis, particularly because the Omicron infection is associated with milder symptoms secondary to the effect of vaccination and viral mutation.

## 10. Future Directions 

As we adapt to virus variants, the next challenge is to encourage bivalent booster vaccine uptake and counter complacency as restrictions ease. Evaluation of the need for additional boosters and the optimum timing will be key. Catching up with the backlog of deferred endoscopic procedures will also be a priority. As time passes, we will take stock of the impact of delayed endoscopy on cancer detection and outcomes. Whilst lockdowns are likely now a remnant of the past, telehealth, non-invasive methods of disease assessment, the transition to subcutaneous therapies, and point of care testing will continue to expand. These lessons learnt will be valuable in the case of future outbreaks, as IBD patients have a unique set of concerns and vulnerabilities. The pandemic shows that patients and clinicians can adapt quickly to the everchanging challenges that arise, provided clear and consistent communication is maintained.

## Figures and Tables

**Table 1:** Adjusted odds ratios (95% CI) of acquisition and outcomes of COVID-19 infections stratified by medication from [4,7].

Agent	Acquisition	Hospitalisation	Severe COVID	Death
**Systemic corticosteroids**	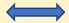 1.64 (1–2.7)	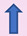 2.4 (1.83–3.31)	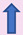 3.41 (2.62–4.64)	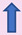 4.77 (3.36–6.77)
**5-ASA**	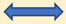 1.89 (1.23–2.93)	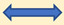 1.02 (0.83–1.26)	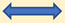 1.03 (0.74–1.83)	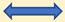 1.09 (0.65–1.82)
**Methotrexate**	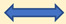 Pooled data immunomodulator 1.55 (0.97–2.48)	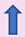 1.26 (1.0–1.57)	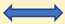 1.04 (0.39–2.81)	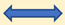 0.79 (0.20–3.08)
**Thiopurines**		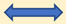 0.96 (0.80–1.15)	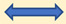 1.34 (0.90–2.0)	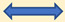 0.93 (0.53–1.65)
**Anti-TNF**	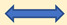 1.06 (0.68–1.71)	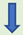 0.58 (0.50–0.69)	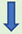 0.50 (0.33–0.78)	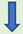 0.44 (0.26–0.76)
**Ustekinumab**	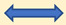 3.16 (0.55–18.7)	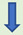 0.44 (0.36–0.54)	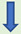 0.43 (0.26–0.71)	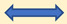 0.55 (0.28–1.11)
**Vedolizumab**	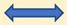 2.31 (1–5.3)	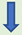 0.66 (0.56–0.76)	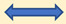 0.72 (0.42–1.24)	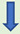 0.50 (0.32–0.78)
**Tofacitinib**	N/A	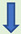 0.48 (0.30–0.76)	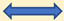 0.50 (0.14–1.86)	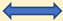 0.83 (0.10–7.11)

Arrows represent risk compared to the general population.

**Table 2:** Impact of medications on vaccine-induced B and T cell responses [2,20,22].

Agent	B Cell Response	T Cell Response
**Systemic corticosteroids**	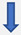	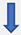
**5-ASA**	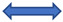	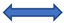
**Immunomodulators**	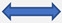	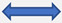
**Anti-TNF**	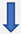	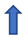
**Ustekinumab**	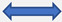	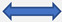
**Vedolizumab**	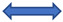	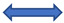
**Tofacitinib**	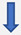	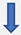

Arrows represent risk compared to the general population.

## Data Availability

Not applicable.
